# Corrosion and Formation of Surface Films on Metals and Alloys

**DOI:** 10.3390/ma19081518

**Published:** 2026-04-10

**Authors:** Johannes Herman Potgieter, David J. Whitefield, Michael O. Bodunrin

**Affiliations:** School of Chemical and Metallurgical Engineering, University of the Witwatersrand, Private Bag 3, WITS, Johannesburg 2050, South Africa; herman.potgieter@wits.ac.za (J.H.P.); david.whitefield@wits.ac.za (D.J.W.)

## Editorial Comment

Corrosion is a multibillion-dollar material degradation issue that affects process industries, transport infrastructure, as well as construction and building activities all over the world. As such, corrosion is one of the major industrial and environmental challenges facing modern society, necessitating huge amounts of maintenance, incurring high replacement costs, and posing safety risks, and therefore requires urgent and constant attention. Various methods can be used to mitigate and reduce corrosion damage. One of the most important approaches involves designing and choosing suitable alloys for particular environments that have the ability to form surface films in these environments, which increase corrosion resistance. This Special Issue focuses on and includes a number of papers in which the authors report research carried out with this aim.

In a study entitled “Corrosion Behaviour of S32101 (1.4162—X2CrMnNiN21-5-1) Stainless Steel in Pulping Liquors”, Banele Siyabonga Kheswa, David Whitefield, Herman Potgieter, and Michael Bodunrin, from the University of the Witwatersrand in South Africa [1], examined the corrosion behavior of S32101 lean duplex stainless steel in environments typical of pulp and paper mills, including in acidic, saline, and industrial liquors such as black, green, and white liquors, as well as in chlorine dioxide bleaching solutions. The results showed that S32101 experienced pitting in chloride-rich solutions and high corrosion rates in highly alkaline white liquor but resisted chlorine dioxide and black liquor. These findings are practically important, guiding material selection for pulp and paper applications, offering a cost-effective alternative to 316L stainless steel and fiberglass, and reducing downtime and maintenance costs.

Two prior studies are directly relevant to the first study [1]. Esteves et al. [2] investigated the corrosion behavior of S31803 lean duplex stainless steel in synthetic and industrial white and green liquors. Their results showed that the synthetic white liquor was more aggressive than the industrial counterpart, highlighting the importance of accurately simulating service environments when evaluating corrosion performance. Kheswa et al. [1] examined the S32101 alloy across a broad range of environments, including synthetic and industrial liquors, to more comprehensively assess its corrosion response under realistic process conditions.

Similarly, Bairi et al. [3] conducted a long-term, 70-day, corrosion study on 2101 lean duplex stainless steel in 3.5 wt% NaCl solution, employing immersion and electrochemical testing techniques. Pitting corrosion resistance increased with l immersion time, which was attributed to the progressive growth and stabilization of the passive film. Although pits were detected after approximately 20 days of immersion, prolonged exposure led to a marked reduction in pit density as the passive film became more compact and stable after approximately 40 days. Beyond this period, no further notable thickening of the passive film was observed, suggesting that a steady-state passive condition was reached. A similar observation was reported in borate buffer solution, a mildly passivating medium, further reinforcing the role of time-dependent passive film maturation in enhancing corrosion resistance.

The topic was further examined in a study entitled “Behavior of YSZ (High Y2O3 Content) Layer on Inconel to Electro-Chemical Corrosion” by Ionut Adomniței, Ramona Cimpoeșu, Daniela Lucia Chicet, Margareta Coteață, Fabian Cezar Lupu, Costică Bejinariu, Liviu Andrușcă, Petronela Paraschiv, Mihai Axinte, Gheorghe Bădărău, and Nicanor Cimpoeșu, from Romania [4]. The authors explored the corrosion resistance of Inconel 718 coated with a high-yttria YSZ ceramic layer applied via atmospheric plasma spraying, using a Ni-Al bond coat for adhesion. The increase in corrosion resistance achieved with this method is crucial for aerospace and energy sectors where components must endure extreme temperatures and corrosive conditions, as ceramic coatings enhance service life and reduce maintenance costs.

Another paper describes the influence of a surface layer on corrosion resistance. Georg Veile, Radhika Hirpara, Simon Lackmann, and Stefan Weihe from Germany discuss their results in the paper entitled “Investigation of Oxide Layer Development of X6CrNiNb18-10 Stainless Steel Exposed to High-Temperature Water” [5]. The researchers investigated the pitting corrosion of type 316 stainless steel under simulated groundwater conditions mimicking spent nuclear fuel canisters, focusing on pH, chloride (Cl^−^), and bisulfide (HS^−^) ions. Their findings showed that chloride and HS^−^ strongly influences passive film breakdown: HS^−^ generally weakens the film but can inhibit corrosion under low-chloride-concentration conditions by forming protective sulfides. These insights are practically important for nuclear waste storage, identifying conditions that minimize localized corrosion as well as increase safety and cost-effectiveness. The researchers combined electrochemical testing, surface analysis, and statistical modeling, making the findings highly relevant for addressing long-term material durability.

The paper entitled “Corrosion of an Additively Manufactured Ti6Al4V Alloy in Saline and Acidic Media” is authored by Hugo Mora-Sanchez, Miguel Collado-Vian, Marta Mohedano, Raúl Arrabal, and Endzhe Matykina from Spain [6]. The authors examined oxide layer growth on X6CrNiNb18-10 stainless steel (AISI 347) under high-temperature water simulating boiling water reactor conditions. The results revealed parabolic growth of the Cr-rich inner layer, cubic growth of the Fe-rich outer layer, and Ni accumulation in the secondary layers, while niobium formed Nb_2_O_5_—a novel observation for this environment. These findings are practically relevant for predicting corrosion behavior and extending the lifetime of nuclear reactor components.

Another use of the effects of material type and production on the material’s corrosion behavior is discussed in the paper entitled “Effect of Sulfide and Chloride Ions on Pitting Corrosion of Type 316 Austenitic Stainless Steel in Groundwater Conditions Using Response Surface Methodology” by Jin-Seok Yoo, Nguyen Thuy Chung, Yun-Ho Lee, Yong-Won Kim, and Jung-Gu Kim from Sungkyunkwan University in Korea [7]. They assessed the corrosion behavior of an additively manufactured alloy produced via direct metal laser sintering in saline (3.5 wt.% NaCl) and acidic environments. The additively manufactured alloys were less resistant to corrosion than conventional alloys in saline media but showed comparable or stronger resistance in acidic solutions, especially after heat treatment. These findings are vital for aerospace and biomedical applications, guiding post-processing strategies for additively manufactured components.

The use of surface films on alloys to mitigate and reduce corrosion damage was investigated in a number of corrosion-related studies.

Rondon-Almeyda et al. [8] examined the localized corrosion behavior of UNS S-32760 super duplex stainless steel under conditions representative of pulp and paper digester environments. The results showed that increasing temperature markedly decreased the pitting resistance of the steel. Corrosion damage intensified at higher temperatures, with hemispherical, open pits reaching depths of approximately 58 µm at 70 °C. Pit initiation was linked to thiosulfate ions that migrated into pits and reduced to H_2_S, promoting corrosion and inhibiting passive film repair. The findings provide valuable guidance for material selection and maintenance in pulp and paper digesters.

In the study by Xiao et al. [9], the corrosion behavior of 2205 duplex stainless steel was examined in chloride solutions containing sulfide ions over a wide range of pH values and chloride concentrations. The results showed that, in low-chloride environments, sulfide ions increased pitting resistance by competitively adsorbing on the steel surface. In high-chloride solutions, however, sulfide exhibited two pH-dependent roles: S^2−^ at pH 11.5 enhances repassivation and reduces pitting, whereas HS^−^ at pH 7 and 9 seriously damages the passive film, lowers the pitting potential, and increases pit density. At pH 4, H_2_S promotes FeS formation, reducing oxide content but maintaining moderate resistance. Pitting preferentially initiates at austenite–ferrite interfaces. These findings offer valuable guidance for managing corrosion in sulfide- and chloride-rich industrial environments.

In addition to pH as well as the type and concentration of ionic species driving corrosion, the protective stability of Cr_2_O_3_ films formed on the surface of stainless steel is strongly influenced by stress states under in-service conditions. Mechanical loading can alter the thickness, compactness, and defect chemistry of the passive film, thereby modifying its susceptibility to localized corrosion. Xiao et al. [10,11] investigated the influence of compressive and elastic tensile stresses on the passivation behavior and pitting mechanism of 2205 duplex stainless steel. Their results showed that, under compressive stress below 60% of the 0.2% proof stress, the thickness and compactness of the passive film increased, resulting in increased resistance to pitting corrosion. However, beyond this threshold, susceptibility to pitting increased due to a reduction in passive film thickness and a corresponding increase in defect concentration. Under elastic tensile stress, the critical threshold was lower, approximately 40% of the 0.2% proof stress. Below this value, the defect concentration decreased and the Cr_2_O_3_ film thickened, enhancing corrosion resistance. When tensile stress exceeded 40% of the proof stress, pitting susceptibility markedly increased as a result of passive film thinning and pit morphology changes, with pits preferentially propagating perpendicular to the direction of applied tensile stress. These findings underscore the importance of stress thresholds in service environments and demonstrate that mechanical loading conditions must be considered when designing methods to mitigate pitting initiation in stainless steel components.

More broadly, the corrosion behavior of passive alloys such as stainless steels cannot be solely explained by the presence or thickness of a Cr_2_O_3_ film. Environmental factors including temperature, aeration conditions, and electrochemical polarization, and the intrinsic crystallographic characteristics of the steel play equally important roles. Monteiro et al. [12] demonstrated that, in caustic soda environments, the corrosion rate under polarized conditions reached as high as 18 mm/year, decreasing to approximately 0.5 mm/year at open-circuit potential under de-aerated conditions. Under these conditions, a dark iron-oxy-hydroxide and nickel oxide layer formed, particularly at 50 °C, indicating that film chemistry strongly evolves with electrochemical state and temperature. Similarly, Ma et al. [13] showed that the intrinsic crystallographic orientation influences the general oxidation behavior of stainless steel. Oxidation more rapidly proceeded on the (111) surface than on the (100) surface during exposure to pressurized water. However, once the oxide thickness reached approximately 15 nm, the oxidation rate reversed on the (111) facet. This behavior was attributed to differences in chromium diffusion kinetics and the availability of space for oxide growth. These observations highlight that passivation is by governed not only oxide thickness but also defect transport, crystallographic orientation, and environmental conditions. Consequently, generalizing corrosion resistance purely in terms of passive film thickness can be misleading if the underlying physicochemical processes controlling film growth and stability are not considered.

Yun et al. [14] examined how top-coat structural parameters influence the thermal stress and durability in Gd–Yb–Y co-doped zirconia (GYYZ)/YSZ double-layer thermal barrier coatings. Experimental testing confirmed that reducing the porosity from 20% to 15% increased the microhardness by 12.8% and extended the thermal cycling life by 87.5%, observations that are valuable for increasing the reliability and service life of high-temperature turbine coatings.

In Romania, Cimpoesu et al. [15] examined the electrochemical corrosion resistance of alumina (Al_2_O_3_) and yttria-stabilized zirconia (YSZ) composite coatings applied to steel substrates using atmospheric plasma spraying. Although porosity influenced behavior—particularly that of the 25% YSZ sample, which showed localized pitting—the overall trend indicated that increasing the YSZ content enhances corrosion resistance. Phase analysis identified α- and γ-alumina and tetragonal zirconia in the sample, consistent with rapid solidification during plasma spraying. From a practical perspective, these results are valuable for industries where steel components operate in acidic environments, such as in chemical processing, energy systems, and infrastructure exposed to acid rain.

The hot corrosion behavior of four nickel-based superalloys—Inconel 718, Udimet 710, Nimonic 75, and Inconel 625—exposed to an aggressive 50/50 wt.% Na_2_SO_4_–V_2_O_5_ molten salt mixture at 900 °C for up to 96 h was studied by Badea and Dombrovschi [16]. Inconel 625 experienced the most severe degradation. The high Mo content of Inconel 625 led to the production of porous, unstable oxide scales. Inconel 718 exhibited significant corrosion associated with Fe-rich oxides and chromium depletion, whereas Udimet 710 showed moderate resistance, benefiting from the Al and Ti phases but still adversely influenced by Mo and W. Nimonic 75 performed best and formed a dense, protective Cr_2_O_3_- and NiCr_2_O_4_-based scale owing to its high Ni and Cr contents and lack of Mo and Fe. These findings are relevant to alloy selection in high-temperature, salt-contaminated environments.

The corrosion behavior of additively manufactured Ti-6Al-4V produced via electron beam melting (EBM) and selective laser melting (SLM) in a 0.9 M NaCl environment was investigated by Metalnikov et al. [17]. Electrochemical testing (OCP, PDP, and EIS) revealed that build orientation played a decisive role in corrosion performance: surfaces built in the XY plane consistently exhibited higher corrosion resistance than those built in the XZ plane, attributed to increased grain boundary density and passive film stability. In addition, the SLM specimens showed slightly stronger corrosion resistance than the EBM specimens. By clarifying the links among additive manufacturing route, microstructure, build orientation, and corrosion behavior, this work provides practical guidance for optimizing additive manufacturing design strategies to increase the durability, safety, and long-term performance of advanced components, especially those of Ti-6Al-4V components operating in saline or physiological environments.

Daroonparvar et al. compared the corrosion and high-temperature oxidation behavior of additively manufactured (AMed) alloys with conventionally manufactured (CMed) counterparts, focusing on how additive-manufacturing-specific microstructural features influence performance in aggressive environments [18]. The materials considered included Al-based alloys, Ti-6Al-4V, 316L stainless steel, and Ni-based superalloys. AMed aluminum alloys often exhibited higher corrosion resistance in chloride media due to their refined silicon networks and fewer intermetallics, although heat treatment reduced this benefit by enhancing micro-galvanic effects. For Ti-6Al-4V, rapid solidification led to the production of α′ martensite and reduced corrosion resistance, effects that could be reversed through appropriate heat treatments. AMed 316L stainless steel generally displayed superior pitting resistance because of its refined cellular structures and absence of MnS inclusions, although porosity could impair passivity. In contrast, AMed Ni-based superalloys tended to experience higher oxidation rates due to segregation and porosity, which could be mitigated using HIP and coatings. These insights support reliable additive manufacturing component design for aerospace, biomedical, and energy applications.

Yan et al. [19] investigated the effect of pulsed laser surface remelting (PLSR) on the wear and corrosion resistance of Ti6Al4V alloy. PLSR decreased the wear rate by 36.2%. Electrochemical tests confirmed the increase in corrosion resistance, with corrosion current density reduced by nearly 60% due to the formation of a dense TiO_2_ passivation film. From a practical standpoint, these improvements directly address critical challenges in implant longevity and integration, reducing risks of wear-induced debris, inflammatory responses, and premature failure.

The articles in this Special Issue, as well as the other papers referenced here from various sources, combine new and novel findings from around the globe and suggest practical solutions to issues in common applications in a variety of corrosive environments, from which practitioners in different fields can benefit. This Special Issue highlights the important role of surface composition, films, and coatings in controlling and mitigating corrosion damage to materials.

## Data Availability

All data related to the articles published in this Special Issue are available on request to the corresponding authors of the articles.
